# E-IMR: e-health added to face-to-face delivery of Illness Management & Recovery programme for people with severe mental illness, an exploratory clustered randomized controlled trial

**DOI:** 10.1186/s12913-018-3767-5

**Published:** 2018-12-12

**Authors:** Titus A. A. Beentjes, Peter J. J. Goossens, Hester Vermeulen, Steven Teerenstra, Maria W. G. Nijhuis-van der Sanden, Betsie G. I. van Gaal

**Affiliations:** 1 Titus Beentjes, IQ Healthcare, Radboud University Medical Center, Radboud Institute for Health Sciences, PO Box 9101, 6500 HB Nijmegen, the Netherlands; 2 Center for Nursing Research, Saxion University of Applied Science, Deventer/Enschede, the Netherlands; 3 Dimence Group Mental Health Care Centre, Deventer, the Netherlands; 4 Department of Public Health, Faculty of Medicine and Health Sciences, University Centre for Nursing and Midwifery, Ghent University, Ghent, Belgium; 5 Department for Health Evidence, Radboud University Medical Center, Radboud Institute for Health Sciences, Group Biostatistics, Nijmegen, the Netherlands; 6 Faculty of Health and Social Studies, HAN University of Applied Sciences, Nijmegen, the Netherlands

**Keywords:** Severe mental illness, E-mental health, Illness management and recovery

## Abstract

**Background:**

E-mental health holds promise for people with severe mental illness, but has a limited evidence base. This study explored the effect of e-health added to face-to-face delivery of the Illness Management and Recovery Programme (e-IMR).

**Method:**

In this multi-centre exploratory cluster randomized controlled trial, seven clusters (*n* = 60; 41 in intervention group and 19 in control group) were randomly assigned to e-IMR + IMR or IMR only. Outcomes of illness management, self-management, recovery, symptoms, quality of life, and general health were measured at baseline (T_0_), halfway (T_1_), and at twelve months (T_2_). The data were analysed using mixed model for repeated measurements in four models: in 1) we included fixed main effects for time trend and group, in 2) we controlled for confounding effects, in 3) we controlled for interaction effects, and in 4) we performed sub-group analyses within the intervention group.

**Results:**

Notwithstanding low activity on e-IMR, significant effects were present in model 1 analyses for self-management (*p* = .01) and recovery (*p* = .02) at T_1_, and for general health perception (p = .02) at T_2_, all in favour of the intervention group. In model 2, the confounding covariate gender explained the effects at T_1_ and T_2_, except for self-management. In model 3, the interacting covariate non-completer explained the effects for self-management (*p* = .03) at T_1_. In model 4, the sub-group analyses of e-IMR-users versus non-users showed no differences in effect.

**Conclusion:**

Because of confounding and interaction modifications, effectiveness of e-IMR cannot be concluded. Low use of e-health precludes definite conclusions on its potential efficacy. Low use of e-IMR calls for a thorough process evaluation of the intervention.

**Trial registration:**

The Dutch Trial Register (NTR4772)

**Electronic supplementary material:**

The online version of this article (10.1186/s12913-018-3767-5) contains supplementary material, which is available to authorized users.

## Background

In spite of the growing interest in e-mental health, evidence for the effectiveness of e-health for people with a severe or serious mental illness (SMI) is limited [1, 2]. Naslund et al. [2] found that e-health interventions for people with SMI have high feasibility and acceptability. Van der Krieke et al. [3] found that people with psychotic disorders were able and willing to engage in e-health, and found larger effects for medication management [3]. However, one should be cautious about drawing conclusions regarding the effectiveness [2, 3]. E-health is used in a wide range of interventions for people with SMI on (1) illness self-management and relapse prevention, (2) promoting adherence to medications and/or treatment, (3) psycho-education, supporting recovery, and promoting health and wellness, and (4) symptom monitoring [2]. E-health interventions make use of personal digital assistance, medication tracking devices, home monitoring systems, smartphone applications, SMS, and web-based interventions [2].

Also in general mental health, e-health approaches show great potential and offer the possibility of expanding access to care while being economically and socially efficient [4]. But e-health interventions in mental health have high attrition rates [5]. The addition of face-to-face contact to e-health is supposed to increase the therapeutic relation and prevent attrition [6]..In the case of people with SMI, e-health components could be added to an evidence-based face-to-face recovery-oriented intervention. Such an intervention is the Illness Management & Recovery programme (IMR) [7]. The IMR is a standardized curriculum-based approach designed to provide people with SMI the information and skills necessary for managing their illnesses effectively and working towards achieving personal recovery goals. In addition to the standard face-to-face delivery of the IMR, an e-health intervention (e-IMR) was designed which follows the IMR-curriculum, and was further developed with the end-users of the intervention [8]. The aim of this study was to explore the effect of the e-IMR for people with SMI who were referred to the Illness Management & Recovery programme.

## Methods

The e-IMR was tested in an exploratory multi-centre cluster randomized controlled trial. According to the Medical Research Council guidance [9], an exploratory trial evaluated an intervention before testing it in a confirmative trial. In this study, a cluster was a subdivision of a mental health institute. The cluster randomization prevented contamination between the intervention and control group participants. Data were collected at baseline, halfway and endpoint. The inclusion period was between January and October 2015. Data collecting lasted until October 2016.

Eligible clusters delivered the IMR-programme as a whole package with an experienced trainer-couple meaning that at least one trainer completed the IMR-total-training organized by the Dutch IMR-network and executed at least the first five modules of the IMR-programme before starting the IMR-programme in the trial.

### Trial monitoring

An employee of the ‘Radboudumc Technology Center – Clinical Studies’ monitored the process of trial administration. The administration of Trial Master Files, both paper as well as computerized files, was independently checked for completeness and accuracy.

### Randomization

A statistician generated a randomization schedule using Statistical Analysis System®, version 9.4. The allocation to the intervention or control group was communicated after the participating institutional board provided their consent to participation. Because of the nature of the intervention, blinding was not possible.

### Sample size

Because of the exploratory character of this study, a power calculation was considered unnecessary.

### Participants

Eligible participants met the following criteria: above 18 years of age; capable of giving informed consent; and meeting the Dutch SMI criteria according to Delespaul [10] (being diagnosed with a psychiatric disorder that causes, and is due to, serious impairments in social and/or occupational functioning which lasts longer than at least a couple of years and necessitates coordinated multidisciplinary care. Persons who were overwhelmed by disability, including dependence, denial, confusion, anger or despair, were excluded from participating.

### Care as usual

All participants, in both the intervention and control group, received care consisting of extensive inpatient and/or outpatient psychiatric treatment including case management. They also received the IMR-programme, which was provided in weekly, 2-h, face-to-face group sessions according to the Dutch version of the IMR 3.0 programme [11] using the hard-copy version of 11 modules.

### Intervention

On top of this care as usual, participants in the intervention group had the opportunity to use the e-IMR intervention [8]. The e-IMR intervention started with a ‘welcome page’ explaining the use of e-IMR and leading participants to the 11 modules. The e-IMR intervention included the same fill-in forms as in the hard-copy version of the IMR-programme. E-IMR added illustrative videos showing peer testimonials to encourage participants to talk more freely about themselves and to take steps in their recovery process. E-IMR also added problem-solving forms at the end of each module, registration of successful coping strategies, and a symptom-monitoring page.

The e-IMR was introduced to the trainers and participants of the intervention group by the first researcher in the second group session. Individuals who did not provide informed consent were allowed to join the e-IMR without participating in the research. The trainer-couples were supported in learning how to support participants in the use of e-IMR; how to install e-IMR on a computer in the session room and how to use e-IMR during the sessions.

In e-IMR, the registration forms of successful coping strategies and the symptom-monitoring page were introduced after the second module ‘practical facts about mental illnesses’. Weekly emails with a link to the e-IMR platform led the participants to the symptom-monitoring page. After closing each module, one of the trainers gave feedback to the participants via the platform and guided the participants to the next module.

### Data collection

Data were collected in face-to-face interviews by the researcher or a researcher assistant at three time points: at baseline, a week before starting the IMR-programme (T_0_); halfway, after completing the 5th module (T_1_); and endpoint, at least a week after finishing the IMR-programme (T_2_). The data were recorded on paper and later transferred into a LimeSurvey® [12] database. The original recorded data as well as the transferred were double-checked for accuracy and completeness.

### Outcome measures

At baseline, independent demographic and clinical characteristics were recorded. At all three time points, six dependent outcome domains were gathered.

At T_0_ the following participant characteristics were collected: age, gender, physical comorbidities, treatment history, cultural background, social economic status, education level, computer/Internet availability and use. At T_0_, the participant’s case manager provided their diagnostic classification according to the Diagnostic and Statistical Manual of Mental Disorders, 4th edition.

The participant’s ability to manage their illness was measured with the consumer version of the **Illness Management & Recovery Scales** (IMRS), consisting of 15 items [13]. The response anchors, on a five-point Likert scale (1–5) vary depending on the item. The IMRS total-up score ranged between 15 and 75. The IMRS’ Cronbach’s alpha is .55–.83 [14–17].

The participants’ self-management ability, which refers to the individual’s knowledge, skill and confidence for managing his/her own health and healthcare, was measured with the **Patients Activation Measure** (PAM-13) [18], consisting of 13 items. The response anchors on a five-point scale, vary from not applicable (0), ‘strongly disagree’ (1) to ‘strongly agree’ (4). The term ‘doctor’ in the items five and six was explained as their mental health clinician, which includes a nurse and/or case manager. Raw scores were transformed into standardized activation scores ranging between 0 and 100. The PAM-13’s Cronbach’s alpha is .84–.88 [19–22].

**The Mental Health Recovery Measure** (MHRM) assessed the participants’ progress in their recovery process. The MHRM consists of 30 items with response anchors, on a five-point scale, varying from ‘strongly disagree’ (0) to ‘strongly agree’ (4), and ‘neutral’ (2) in between [23]. The MHRM total-up scores ranged between 0 and 120. The MHRM’s Cronbach’s alpha is .93 [24].

The participants estimated the level of burden of symptoms they experienced using the **Brief Symptom Inventory** (BSI), consisting of 53 items [25]. The response anchors, on a five-point scale, vary from ‘not at all’ (0) to ‘extremely’ (4). The mean BSI scores ranged between 0 and 4. A negative time trend for the BSI means a reduced level of burden. The BSI’s Cronbach’s alpha is .96 [26].

The participants’ subjective satisfaction with life was measured with the **Manchester Short Assessment of quality of life** (MANSA), consisting of 12 items [27]. The response anchors on a seven-point scale vary from ‘couldn’t be worse’ (1) to ‘couldn’t be better’ (7). The mean MANSA score ranged between 1 and 7. The MANSA’s Cronbach’s alpha is .81 [28].

The participants’ general health status was measured with the **Rand 36-item Health Survey** (Rand-36), consisting of eight subscales: physical functioning (Rand-PF), social functioning (Rand-SF), role limitations due to a physical (Rand-RLPP) and an emotional problem (Rand-RLEP), mental health (Rand-MH), vitality (Rand-V), pain (Rand-P), and general health perception (Rand-GHP) [29]. The response anchors vary between yes/no to Likert scales with three, five, and six options. Raw scores of all the concepts were transformed into scores ranging between 0 and 100. The Cronbach’s alpha of Rand-36’s eight concepts are .71 and .92 [30].

The extent of participants’ activity on the e-IMR platform was determined by counting the number of completed modules and number of log-ins. An e-IMR user is identified by having completed at least module one or having logged in at least five times. Users were regarded as having had the opportunity to benefit from the e-IMR.

As in other studies on IMR [31], participants who attended the face-to-face IMR programme sessions less than 50% were considered to be non-completers. In our study, this resembles stopping the IMR programme before T_1_.

### Statistical methods

The Statistical Package for the Social Sciences®.23 [32] was used to carry out the analyses. Mixed model multilevel regression analyses were used to examine the main effects on the outcome measures, taking into account clustering of participants and repeated measures. This method automatically uses the ‘missing at random’ assumption to handle missing data. Random effects on cluster, trainer-couple, and individual participants nested within the cluster were included in the model. Model 1 included fixed main effects for time trend and group. The analyses were executed according to the intention-to-treat principle to prevent bias caused by the loss of participants [33] and to reflect the normal practice [34] of high attrition rates in treatments of people with SMI [7] and e-health [5].

Post hoc analyses of effect differences were performed to control for covariates. We considered the covariate gender to be a potential confounder because of its known differences in exposure and reactions to stress and health [35]. The covariate was included in model 2, controlling for confounding time trend effects.

In model 3, covariates were included that were expected to interact with the effect differences.

Non-completion of the face-to-face IMR-programme sessions was expected to interact with the effects because being a non-completer is correlated to lower functioning; for instance, lower social functioning [36] and higher emergency room visits and hospitalization [37]. In addition, we searched for correlations in T_0_ scores between the groups of completers and non-completers.

Because of the known low adherence-rate to Web-based interventions [5], additional subgroup analyses were performed within the intervention group to investigate whether actual use compared to non-use of the e-IMR leads to outcome differences. Thus in model 4, two groups of e-IMR users and non-users were included according to the aforementioned adherence measurement.

## Results

### Participant flow

**Fig. 1:**
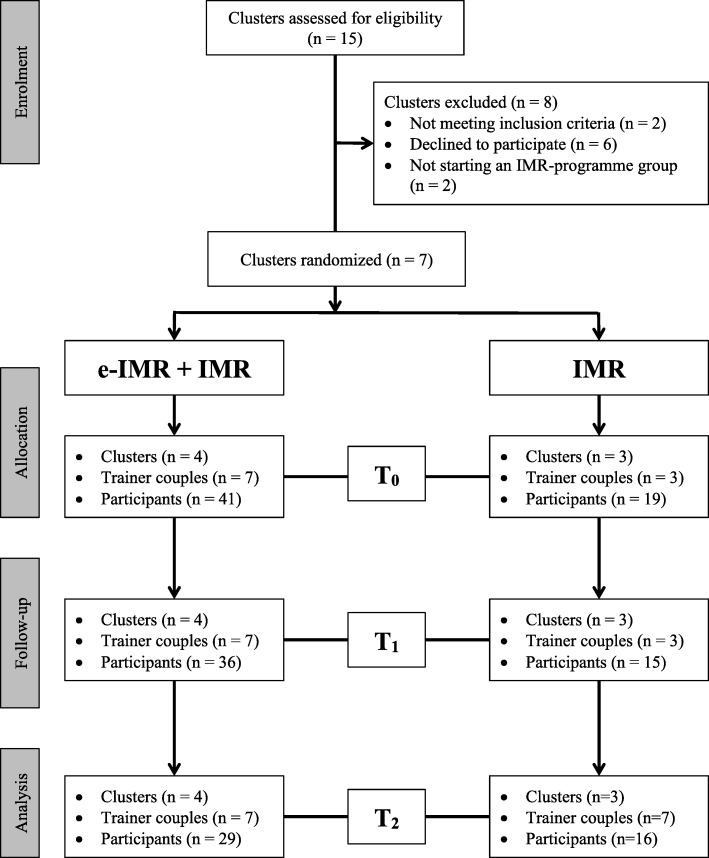
Participants flow diagram through the study

Nine institutions with potentially 15 clusters were screened for eligibility. Two clusters were not eligible because they did not deliver the IMR-programme as a whole. Two clusters did not start an IMR-programme group. Four clusters declined because of organizational problems. Seven clusters were included: four were allocated to the intervention group and three to the control group. In three intervention clusters, a second trainer-couple started a second IMR group. So in total, ten IMR-programme groups (seven in the intervention and three in the control group) trained 60 participants: 41 in the intervention and 19 in the control group (see Fig. 1).

Table 1 shows baseline characteristics and distribution over the two groups. The characteristics ‘gender’ and ‘inpatients/outpatients’ were unequally distributed over the groups, *p* = .002 and *p* = .02 respectively.

**Table 1:** Demographic and clinical characteristics at baseline per group

Variables	Intervention group	Control group
	n (% within group)	n (% within group)
	Mean (SD)	Mean (SD)
Participants	41	19
Age	46.9 (11.6)	40.7 (10.6)
Gender^**^		
Female	30 (73.2)	6 (31.6)
Male	11 (26.8)	13 (68.4)
Diagnoses		
Psychotic disorders	14 (34.1)	6 (31.6)
Mood/anxiety disorders	15 (36.6)	10 (52.6)
Other disorders	12 (29.3)	3 (15.8)
Global Assessment of Functioning	50.86 (8.2)	49.8 (10)
Having a somatic comorbidity	23 (56.1)	7 (36.8)
Having a psychiatric comorbidity	27 (65.9)	11 (57.9)
Treatment history		
Years ago since first treatment	17.15 (12.3)	16.17 (9.9)
Number of admissions	4.15 (3.9)	3.94 (3.3)
Never admitted	7 (17)	2 (10.5)
Cultural Background		
Dutch	37 (90.2)	19 (100)
Turkish, Maroc, Surinam, or English	4 (9.8)	0 (0)
In/outpatients^*^		
Independent living	30 (73.2)	8 (42.1)
Supported housing	11 (26.8)	11 (57.9)
Netto income		
≤ Minimal income	31 (75.6)	16 (84.2)
> Minimal income	10 (24.4)	3 (15.8)
Highest graduated education		
≤ Middle school	26 (63.4)	12 (63.2)
≥ High school	15 (36.6)	7 (36.8)
Computer availability / usage		
I don’t have a computer/laptop	8 (19.5)	3 (15.8)
I never use a computer/laptop	6 (14.6)	2 (10.5)

*Abbreviations*: *n* number; *SD* Standard Deviation;

^*^&^**^: significant between group differences ^*^p < .05; ^**^p < .01 (2-tailed)

All ten IMR-programme groups completed the trial. In the intervention group, 12 out of 41 participants were lost in the follow-up measures in the study. We lost five at T_1_ and another seven at T_2_. In the control group, four out of 19 participants were lost in the follow-up at T_1_ and T_2_. We have missing data at T_1_ for one participant in the control group. Participants either refused to be interviewed because of being too burdened by the interviews, or they did not respond to attempts to get in touch with them. Out of the 60 participants, 51 (36 and 15) participants were interviewed at T_1_, and 45 at T_2_ (29 and 16) (See Fig. 1).

Out of the total of 60 participants, eighteen (30%) were identified as a non-completer: participants who attended the face-to-face IMR programme sessions less than 50%. Eight participants (20%) in the intervention group and ten participants (58%) in the control group were non-completers, which differed significantly (*p* = .01). Of these non-completers, 14 participants entered the intention-to-treat analyses, eight in the intervention group and six in the control group at T_1_, and seven in both groups at T_2_.

**Fig. 2:**
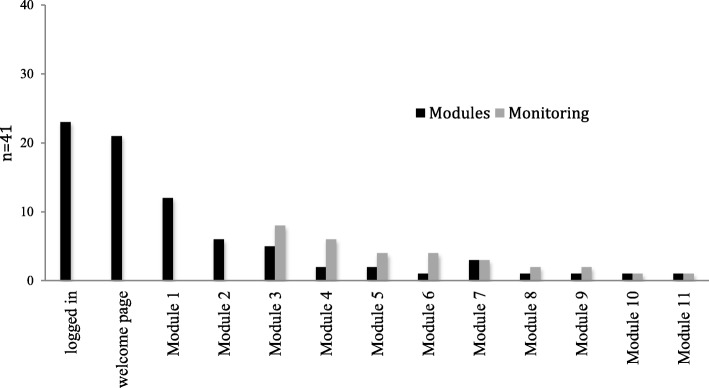
Number of participants active on the e-IMR platform within the intervention group

Out of the 41 participants in the intervention group, 23 (56.1%) logged in on the e-IMR platform, twelve of whom completed the first online module and eight of whom visited the symptom-monitoring page (See Fig. 2). In total, 14 (34.1%) participants were identified as e-IMR users.

### Outcomes and estimation

The mean scores and standard deviations of the outcomes in both groups are presented in the Additional file 1. Since the random effect of cluster was zero in nearly all the analyses, this factor was excluded from the analyses models. The relevant results of the mixed model analyses are shown in Table 2. In model 1, the participants in the intervention group scored significantly higher compared to the control group for the measures PAM-13 (*p* = .01), MHRM (*p* = .02), and Rand-RLEP (*p* = .03) at T_1_, which faded at T_2_. At T_2_, the effect on the Rand-GHP was significant (p = .02) in favour of the intervention group.

**Table 2:** Mixed Model analyses, effect differences for outcome domains

			Model 1		Model 2					Model 3					Model 4	
			Main group effects		Confounder analyses of covariate gender					Interaction analyses of covariate non-completer					Sub-group analyses within intervention group	
Outcome domains		Parameter	T_1_*Group	T_2_*Group	T_0_*Male	T_1_*Male	T_2_*Male	T_1_*Group	T_2_*Group	T_0_* completer	T_1_*Group	T_1_*Group* completer	T_2_*Group	T_2_*Group* completer	T_1_*e-IMR-users	T_2_*e-IMR-users
Illness management: IMRS		effect	2.55	3.06	4.89	−2.31	−3.26	2.26	2.42	1.57	1.12	.62	2.38	.74	1.89	1.71
		p	.13	.07	.00^**^	.19	.08	.19	.17	.38	.70	.86	.41	.84	.32	.44
Self-management: PAM-13		effect	7.95	3.90	4.40	.75	1.76	8.71	5.04	1.52	*17.72*	*−15.13*	6.80	−4.81	−.73	3.03
		p	.01^*^	.22	.19	.82	.62	.01^*^	.14	.67	.00^*^	.03^*^	.21	.48	.81	.40
Recovery: MHRM		effect	7.23	5.06	12.69	−7.02	−3.55	5.35	4.42	6.89	13.36	−10.90	8.09	−3.85	3.27	.84
		p	.02^*^	.11	.00^**^	.03^*^	.28	.10	.19	.13	.01^*^	.10	.14	.57	.32	.82
Symptoms: BSI		effect	−.07	−.13	−.60	.28	.31	−.02	−.07	−.13	.08	−.12	.06	−.24	−.18	−.22
		p	.58	.30	.00^**^	.03^*^	.02^*^	.90	.62	.47	.72	.66	.77	.39	.19	.17
Quality of Life: MANSA		effect	.15	.11	.37	−.22	−.36	.10	.00	.10	.49	−.58	−.04	.24	.10	.12
		p	.35	.52	.07	.18	.04^*^	.57	.99	.65	.08	.09	.90	.50	.57	.55
General Health Status:	Rand-PF	effect	6.61	5.21	19.87	3.57	−6.59	8.73	3.63	−3.92	13.2	−13.01	6.1	−3.17	−.48	6.78
		p	.16	.27	.00^**^	.45	.18	.07	.46	.58	.11	.20	.46	.76	.93	.26
	Rand-SF	effect	−.02	.93	15.91	−1.24	−14.26	−1.67	−2.24	1355	2.04	−7.55	−3.39	5.04	.35	9.07
		p	1.00	.88	.02^*^	.13	.04^*^	.80	.74	.04^*^	.85	.58	.76	.71	.96	.24
	Rand-RLPP	effect	9.47	7.88	29.85	−3.43	−13.61	9.98	4.68	−11.85	29.73	−32.31	11.28	−6.33	−6.56	4.99
		p	.39	.48	.01^*^	.76	.26	.39	.69	.36	.12	.17	.55	.79	.59	.73
	Rand-RLEP	effect	−25.58	9.47	29.18	−9.99	−17.38	− 21.33	11.43	8.18	−28.77	5.24	−21.27	44.85	−18.90	23.40
		p	.03^*^	.43	.01^**^	.46	.22	.09	.37	.47	.16	.84	.31	.08^*^	.15	.13
	Rand-MH	effect	−2.40	−1.09	15.11	−6.73	−5.80	−3.85	−2.17	8.07	−.50	−5.33	−4.94	5.65	2.24	−.83
		p	.57	.80	.00^**^	.13	.21	.39	.64	.13	.95	.56	.50	.54	.61	.87
	Rand-V	effect	−.26	.91	15.11	−3.18	−7.04	.55	.35	8.76	14.76	−22.9	8.6	−8.36	−7.62	−9.42
		p	.96	.85	.00^**^	.53	.19	.92	.95	.11	.07	.03^*^	.30	.42	.11	.09
	Rand-P	effect	1.63	−3.46	21.37	2.74	−1.11	6.01	−3.74	−10.34	−4.51	2.84	−6.01	.68	2.14	−1.73
		p	.82	.63	.00^**^	.72	.21	.42	.62	.19	.72	.85	.63	.97	.80	.28
	Rand-GHP	effect	7.84	1.10	15.21	−2.91	−13.97	7.13	5.31	1.65	9.28	−3.13	9.44	3.43	−3.01	5.26
		p	.07	.02^*^	.00^**^	.50	.00^**^	.10	.23	.76	.22	.73	.21	.71	.53	.35

*BSI* Brief Symptom Inventory, *e-IMR* e-health application to Illness Management & Recovery programme, *IMRS* Illness Management & Recovery Scales, *MANSA* Manchester Short Assessment of Quality of Life, *MHRM* Mental Health Recovery Measure, *p* p-value, *PAM* Patient Activation Measure, *Rand-GHP* Rand General Health Perception, *Rand-MH* Rand Mental Health, *Rand-P* Rand Pain, *Rand-PF* Rand Physical Functioning, *Rand-SF* Rand Social Functioning, *Rand-RLEP* Rand Role Limitation due to Emotional Problems, *Rand-RLPP* Rand Role Limitation due to Physical Problems, *Rand-V* Rand Vitality; ^*^p-value < .05; ^**^p-value < .01

### Post hoc analyses

In model 2, the analyses accounting for the covariate gender showed that the significant effects above could be explained by confounding except for the remaining effect for PAM-13 (p = .01) at T_1_. At T_0_, male participants scored significantly higher on nearly all the measures except for the PAM-13. The same exception occurred in the time trends, but contrarily in favour of female participants.

**Fig. 3:**
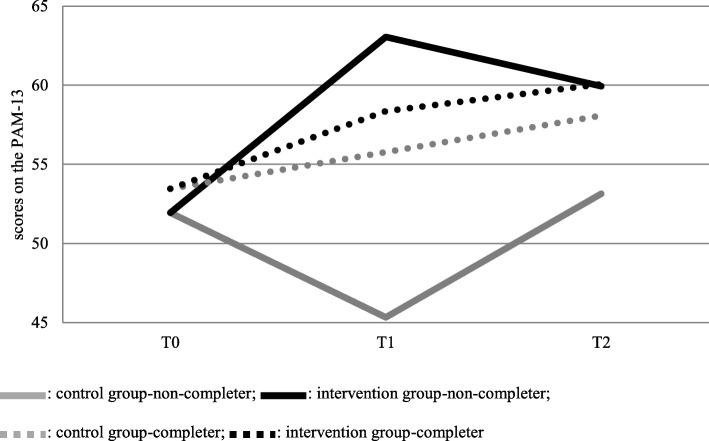
The course of PAM-13 scores in analysis with covariate non-completer

In model 3, the analyses showed that the interaction of the covariate non-completer was significant for the measures: PAM-13, (p = .03) and Rand-V (p = .03) at T_1_, which faded at T_2_. As an illustration of the interaction, the graphic in Fig. 3 shows the scores for the PAM-13, which resembles the scores of the Rand-V. We did not find significant correlations in PAM-13 scores at T_0_ between the completers and non-completers (*p* = .77).

In model 4, the subgroup analyses within the intervention group between the groups of e-IMR users and non-users showed no significant effect differences at T_1_ and T_2_.

### Harm

No serious adverse events were reported during the trial.

## Discussion

This study shows significant differences in main effects for the parameters self-management (PAM-13), recovery (MHRM), and role limitation due to emotional problems (Rand-RLEP) in favour of the intervention group at T_1_, which faded at T_2_. At T_2_, a significant effect for general health perception (Rand-GHP) occurred, also in favour of the intervention group.

Post hoc analyses showed that the confounder gender explained the effects for recovery and role limitation due to emotional problems at T_1,_ and for general health perception at T_2_. The confounding effects of gender were based on three types of differences: first, the baseline distribution showed significantly more females in the intervention group; second, at T_0_ males scored significantly higher on most of the measures; and third, time trends were in favour of female participants. In general, women do differ from men in a number of ways; for instance, exposure and reactions to stress [35], needs and care [38, 39], and coping styles [40]. With regard to coping styles, women could benefit more from a problem-solving-focused intervention and men from an emotion-focused one [41]. IMR, with its emphasis on learning how to manage an illness in a context of pursuing recovery goals [42], has a greater focus on problem-solving- than on emotional strategies. Therefore, women could have benefitted more from the IMR-programme than men.

Post-hoc analyses showed that the confounder gender did not explain the effects for the parameter self-management. Also in studies with people with diabetes II [43] and other chronic illnesses [44], no relations were found between gender and self-management, measured by the PAM-13.

The interaction covariate non-completer significantly modified the effect for the parameter self-management (PAM) and vitality (Rand-V) such that a large intervention effect was seen in the non-completers and a small effect in the completers. Apparently, stopping the IMR-programme was based on differences in their improvements. In this study, improvements in conditions of people who dropped out of the IRM-programme were unequally distributed over the groups, which modified the effects. The unlikeliness of the effects is confirmed by the subgroup analyses within the intervention group which showed no significant effect differences between the groups of e-IMR users and non-users.

A last issue to discuss is the low use of the e-IMR platform by the participants in the intervention group which resulted in a minor contrast in the treatments provided to the participants in the intervention and control group and further calls into question the validity of ascribing the effects observed to the e-IMR. The modest use of the e-IMR matches with 6% of consumers using e-health in general mental health in the year of this study [45].

A number of limitations should be noted. Unfortunately, the planned sample size was not achieved and a lower number of participants entered the control group. This might have caused the unequal distribution of some covariates. Due to the small sample, we could not control for more than one covariate in the mixed models without risking overfitting. Notwithstanding the small sample, a number of non-completers did not withdraw from the study. The overall non-completer rate of 30% is similar to other IMR studies [7]. Therefore, the intention-to-treat analyses resemble normal practice.

## Conclusion

Finally, this study precludes definite conclusions on the potential efficacy of e-health for people with SMI. This leaves us with many questions about the barriers and facilitators of the e-IMR intervention and its implementation. Against the backdrop of the great promise of e-mental health [46], the modest use of the e-IMR platform might be an interesting outcome which needs to be further investigated. Before deciding how to continue studying the effectiveness of e-IMR, we will investigate barriers and facilitators of the e-IMR and its implementation.

## Additional files

Additional file 1: Mean scores and standard deviation of the outcome domains per group at baseline (T_0_), halfway (T_1_) and post treatment (T_2_) (DOCX 29 kb)

## Abbreviations

BSI: Brief Symptom Inventory
e-IMR: e-health application to Illness Management & Recovery programme
IMR: Illness Management & Recovery programme
IMRS: Illness Management & Recovery Scales
MANSA: Manchester Short Assessment of Quality of Life
MHRM: Mental Health Recovery Measure
PAM-13: Patient Activation Measure
Rand-GHP: Rand General Health Perception
Rand-MH: Rand Mental Health
Rand-P: Rand Pain
Rand-PF: Rand Physical Functioning
Rand-RLEP: Rand Role Limitation due to Emotional Problems
Rand-RLPP: Rand Role Limitation due to Physical Problems
Rand-SF: Rand Social Functioning
Rand-V: Rand Vitality
SMI: Severe (or Serious) Mental Illness
T_0_: Time point 0, baseline;
T_1_: Time point 1, halfway, after completing the 5th module
T_2_: Time point 2, endpoint, at least a week after finishing the IMR-programme
