# Supplementary material for: E-IMR: e-health added to face-to-face delivery of Illness Management & Recovery programme for people with severe mental illness, an exploratory clustered randomized controlled trial
Source: BMC Health Serv Res. 2018 Dec 12;18:962. doi: 10.1186/s12913-018-3767-5 (PMC6292084; doi:10.1186/s12913-018-3767-5)
Supplement: Supplementary file 1 — Mean scores and standard deviation of the outcome domains per group at baseline (T0), halfway (T1) and post treatment (T2) (DOCX 29 kb) [file 12913_2018_3767_MOESM1_ESM.docx]

| **Additional file** Mean scores and standard deviation of the outcome domains per group at baseline (T_0_), halfway (T_1_) and post treatment (T_2_) | | | | | |
| --- | --- | --- | --- | --- | --- |
|  | | Time point | T_0_ | T_1_ | T_2_ |
| Measure | | Group | Mean (SD) | Mean (SD) | Mean (SD) |
| Illness management: IMRS | | Control | 53.1 (7.5) | 53.8 (7.3) | 54.6 (5.9) |
|  |  | Intervention | 51.6 (6.3) | 55.1 (6.0) | 56.7 (5.9) |
| Self-management: PAM-13 | | Control | 54.4 (13.5) | 52.9 (9.9) | 58.3 (15.5) |
|  |  | Intervention | 52.5 (10.0) | 59.1 (14.0) | 60.4 (13.1) |
| Recovery:  MHRM | | Control | 71.4 (14.9) | 7.9 (15.7) | 77.2 (17.4) |
|  |  | Intervention | 66.6 (16.4) | 74.0 (14.7) | 79.2 (15.9) |
| Symptoms:  BSI | | Control | 1.0 (0.7) | 0.9 (0.6) | 0.9 (0.6) |
|  |  | Intervention | 1.1 (0.7) | 0.9 (0.6) | 0.8 (0.7) |
| Quality of Life:  MANSA | | Control | 4.3 (0.7) | 4.4 (0.8) | 4.4 (0.6) |
|  |  | Intervention | 4.2 (0.9) | 4.6 (0.8) | 4.6 (0.7) |
| General Health Status: | Rand-PF | Control | 78.4 (26.0) | 73.3 (31.6) | 80.3 (22.3) |
|  |  | Intervention | 72.3 (24.6) | 77.2 (25.0) | 78.6 (23.5) |
|  | Rand-SF | Control | 61.2 (23.9) | 64.2 (23.1) | 60.2 (22.9) |
|  |  | Intervention | 61.0 (25.3) | 64.2 (23.9) | 61.6 (26.3) |
|  | Rand-RLPP | Control | 55.3 (45.3) | 51.7 (45.8) | 57.8 (49.8) |
|  |  | Intervention | 45.7 (44.0) | 53.5 (46.0) | 54.3 (44.9) |
|  | Rand-RLEP | Control | 36.8 (45.7) | 62.2 (37.5) | 43.8 (33.8) |
|  |  | Intervention | 44.7 (39.2) | 40.7 (39.9) | 60.9 (44.6) |
|  | Rand-MH | Control | 56.0 (20.9) | 58.7 (17.3) | 59.0 (17.6) |
|  |  | Intervention | 56.7 (18.2) | 58.4 (19.0) | 61.8 (21.0) |
|  | Rand-V | Control | 47.4 (23.1) | 50.3 (24.5) | 50.3 (19.4) |
|  |  | Intervention | 48.7 (19.5) | 51.9 (16.5) | 53.8 (17.7) |
|  | Rand-P | Control | 71.9 (27.3) | 70.1 (32.9) | 75.1 (23.7) |
|  |  | Intervention | 61.7 (27.8) | 67.7 (28.2) | 66.9 (29.6) |
|  | Rand-GHP | Control | 55.5 (19.3) | 49.7 (16.0) | 52.8 (16.9) |
|  |  | Intervention | 46.6 (18.0) | 51.0 (21.0) | 57.1 (17.1) |
| BSI: Brief Symptom Inventory; IMRS: Illness Management & Recovery Scales; MANSA: Manchester Short Assessment of Quality of Life; MHRM: Mental Health Recovery Measure; MHRM-SE: MHRM self empowerment; MHRM-LN: MHRM learning and new potentials; MHRM-S: MHRM spirituality; PAM: Patient Activation Measure; Rand-PF: Rand-GHP: Rand general health perception; Rand-MH: Rand mental health; Rand-P: Rand pain; Rand-PF: Rand physical functioning; Rand-SF: Rand social functioning; Rand-V: Rand vitality; SD: standard deviation. | | | | | |
